# The iron-sulfur cluster is essential for DNA binding by human DNA polymerase ε

**DOI:** 10.1038/s41598-022-21550-4

**Published:** 2022-10-19

**Authors:** Alisa E. Lisova, Andrey G. Baranovskiy, Lucia M. Morstadt, Nigar D. Babayeva, Elena I. Stepchenkova, Tahir H. Tahirov

**Affiliations:** 1 Fred and Pamela Buffett Cancer Center, Eppley Institute for Research in Cancer and Allied Diseases, University of Nebraska Medical Center, Omaha, NE 68198 USA; 2 Present Address: Department of Genetics and Biotechnology, Vavilov Institute of General Genetics, Saint-Petersburg Branch, Saint-Petersburg State University, Russian Academy of Sciences, St. Petersburg, Russia

**Keywords:** Biochemistry, Structural biology

## Abstract

DNA polymerase ε (Polε) is a key enzyme for DNA replication in eukaryotes. Recently it was shown that the catalytic domain of yeast Polε (Polε_CD_) contains a [4Fe-4S] cluster located at the base of the processivity domain (P-domain) and coordinated by four conserved cysteines. In this work, we show that human Polε_CD_ (hPolε_CD_) expressed in bacterial cells also contains an iron-sulfur cluster. In comparison, recombinant hPolε_CD_ produced in insect cells contains significantly lower level of iron. The iron content of purified hPolE_CD_ samples correlates with the level of DNA-binding molecules, which suggests an important role of the iron-sulfur cluster in hPolε interaction with DNA. Indeed, mutation of two conserved cysteines that coordinate the cluster abolished template:primer binding as well as DNA polymerase and proofreading exonuclease activities. We propose that the cluster regulates the conformation of the P-domain, which, like a gatekeeper, controls access to a DNA-binding cleft for a template:primer. The binding studies demonstrated low affinity of hPolε_CD_ to DNA and a strong effect of salt concentration on stability of the hPolε_CD_/DNA complex. Pre-steady-state kinetic studies have shown a maximal polymerization rate constant of 51.5 s^−1^ and a relatively low affinity to incoming dNTP with an apparent *K*_*D*_ of 105 µM.

## Introduction

DNA polymerase ε (Polε) is one of the main eukaryotic replicases responsible for leading-strand synthesis during genome duplication^1–3^. It belongs to the B-family of DNA polymerases, which also contains Polδ, Polα, and Polζ. Human Polε (hPolε) consists of four subunits: the catalytic subunit (p261) and the accessory subunits p59, p17, and p12, listed in order from bigger to smaller. The p261 is composed of two duplicated exonuclease/polymerase domains, the first of which is active, while the second is inactive and plays an important structural role^4–6^. In the recently reported structure of the yeast Polε holoenzyme, the small subunits p17 and p12 are tethering the two lobes of p261^7^. Instability of hPolε holoenzyme results in replication stress, tumorigenesis, and developmental abnormalities^8,9^.

The Polε catalytic subunit contains three cysteine motifs: CysA, CysB, and CysX^10,11^. The first two are located at the extreme C-terminus, which interacts with a B-subunit (p59 in humans). The recently discovered CysX motif is located in the N-terminal domain and responsible for DNA polymerase and exonuclease activities. The first report about the presence of a [4Fe-4S] cluster in yeast Polε is dated to 2011, when the cluster location was attributed to a CysB motif^12^. This cluster position was not confirmed by subsequent work of our group^13^, which is consistent with the crystal structure of the human Polε subcomplex p59-p261 (2142–2286) showing zinc in CysA and CysB^14^. In 2014, Jain et al*.* discovered the CysX motif and demonstrated its importance in the [4Fe-4S] cluster coordination and in DNA polymerase activity of yeast Polε^10^. Later, the presence of a iron-sulfur cluster in the CysX motif of yeast Polε was confirmed using a structural approach^11^. Recently it was shown that the iron-sulfur cluster of yeast Polε is redox active and its oxidation affects DNA polymerase activity^15^. Despite these advances, the mechanism of DNA synthesis regulation by the iron-sulfur cluster is still unclear and its role in Polε interaction with DNA has not been studied so far.

In this work, we showed that the [4Fe-4S] cluster is critical for template:primer binding by human Polε. Two point mutations that disrupt cluster coordination abrogate hPolε interaction with DNA, resulting in the loss of DNA polymerase and exonuclease activities. We also analyzed the outcome of the protein expression system on the level of iron and DNA-binding properties of hPolε samples. In addition, we conducted pre-steady-state kinetic and binding studies of hPolε at near-physiological salt concentration.

## Results

### The iron-sulfur cluster is important for hPolε interaction with DNA

**Figure 1:**
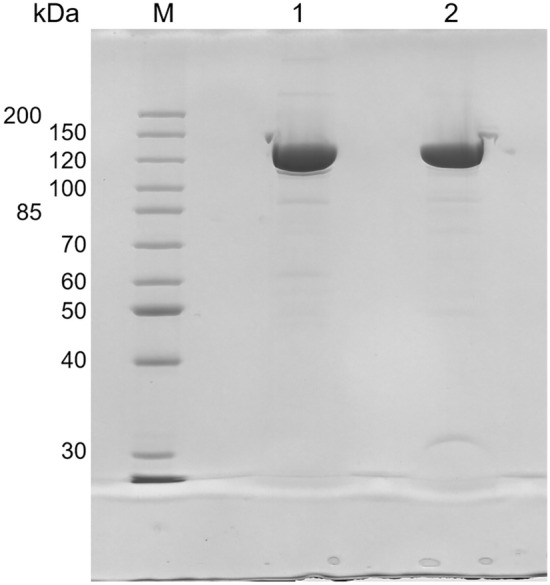
Analysis of purity of hPolε_CD_ samples. Samples were separated by 8% SDS-PAGE and stained by Coomassie Brilliant Blue R-250. M–markers; lane 1–hPolε_CD_(insect); lane 2–hPolε_CD_ (*E.coli*).

The catalytic domain of human DNA polymerase ε (hPolε_CD_; residues 28-1194) containing only the CysX motif was expressed in *Escherichia coli* and purified to near homogeneity (Fig. 1). The concentrated sample displayed a brownish color, a characteristic of proteins containing an iron-sulfur cluster^10,11^. Interestingly, the same protein overexpressed in insect cells and purified in the same way (purity is shown in Fig. 1) was almost colorless. Analysis of the iron content revealed that the hPolε_CD_ sample expressed in insect cells has an 8.6-fold-lower iron level than the one expressed in *E. coli* (Table 1). This result indicates that, upon using the routine protocol, insect cells cannot efficiently incorporate the iron-sulfur cluster into a heterologous protein during its overexpression.

**Table 1:** DNA-binding activity of hPolε_CD_ samples correlates with their iron content.

Expression system	Iron content ^a^%	Active molecules^b^ %
insect	7.3 ± 0.93	8.3 ± 0.7
*E. coli*	63 ± 7.1	51.2 ± 4.1

^a^The iron content of four atoms per protein molecule was taken for 100%.

^b^The molecules showing DNA binding activity.

Data are presented as mean ± SD.

**Figure 2:**
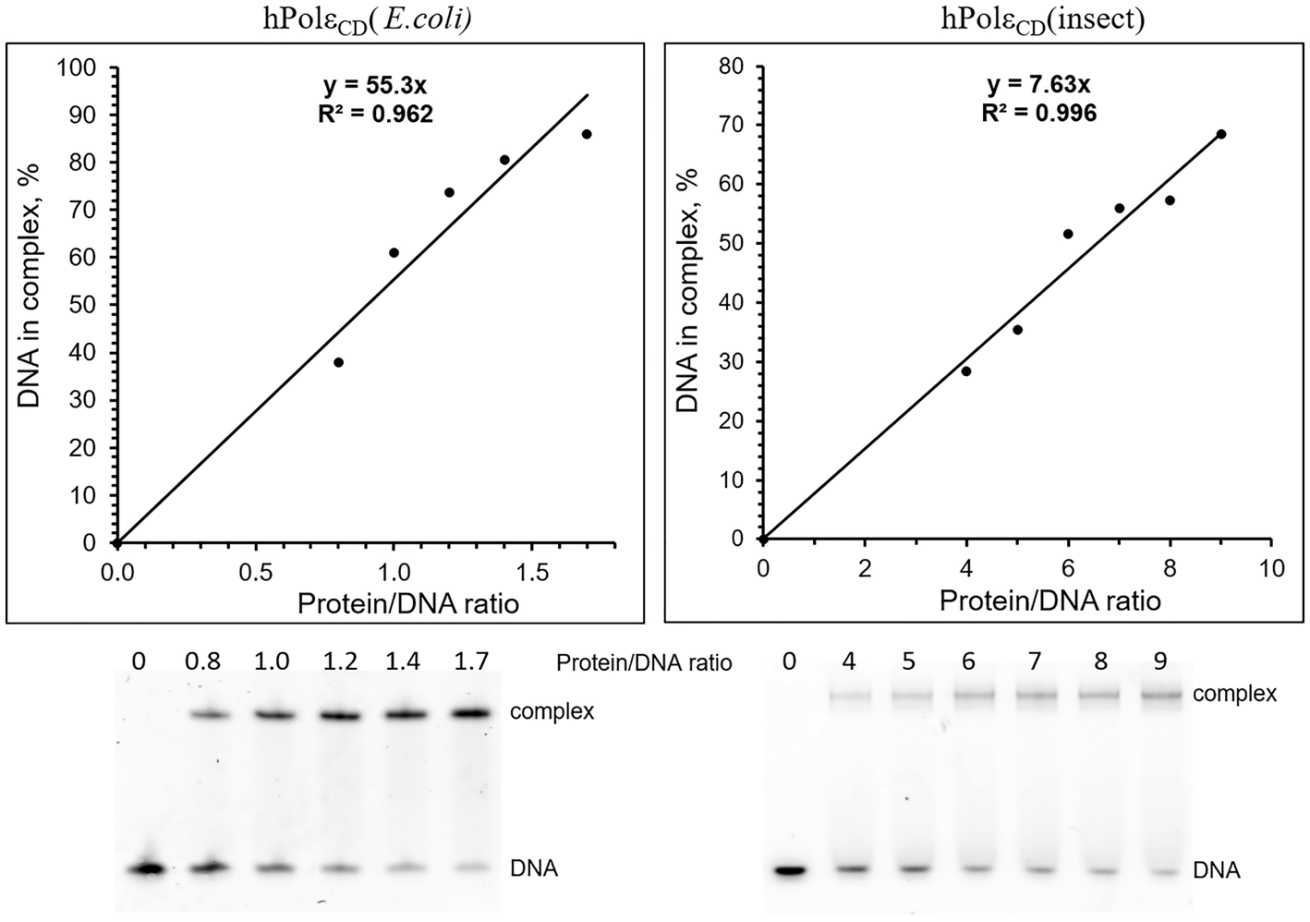
Analysis of the level of active molecules by EMSA. The Cy3-labeled DNA (0.5 µM) was incubated for 5 min with varying amount of protein. The products were separated by electrophoresis in 5% acrylamide gel and visualized by Typhoon FLA 9500. The percent of DNA in the complex is plotted against the protein/DNA ratio, and the generated trend line shows the percent of active molecules.

For both hPolε_CD_ samples, we analyzed the level of molecules possessing DNA-binding activity by using an electrophoretic mobility shift assay (EMSA; Fig. 2). All reactions contained varying concentrations of hPolε_CD_ and 0.5 µM DNA duplex composed of a 15-mer primer annealed to a 20-mer template (Table 2). After 5 min of incubation at room temperature, the products were resolved by a 5% native gel. This approach allows to separate DNA molecules from protein/DNA complexes and calculate the percentage of complexed DNA for each protein concentration. We found that hPolε_CD_ samples obtained after expression in *E. coli* and insect cells have 51% and 8.5% of active molecules, respectively (Table 1). The correlation between the iron content and the level of DNA-binding molecules suggests that the iron-sulfur cluster is important for the interaction of hPolε with DNA. All data described below were obtained using the active enzyme concentration unless otherwise indicated.

**Table 2:** Oligonucleotides used in this study.

Sequence	Description	Application	Length
5′-ATTATGGCAGCTCGGAGTCC^a^	Template	EMSA	20
5′-/Cy3/GGACTCCGAGCTGCC	Primer		15
5′-AATGTTTCTAGGCAGCTCGGAGTCC	Template	kinetic studies	25
5′-/Cy3/GGACTCCGAGCTGCC	Primer		15
5′-/Biotin/AATACATAAGCGCTCCAGGCAAT	Template	Octet K2	23
5′-GCCTGGAGCG/3ddC/	Primer		11

^a^The template regions complementary to a primer are underlined.

### hPolεCD mutant deficient in the [4Fe-4S] cluster displays no DNA interaction and is inactive on the primed template

In order to confirm that the [4Fe-4S] cluster is required for interaction with a template:primer, we obtained the hPolε_CD_ mutant (hPolε_CD_^M^) where two of four conserved cysteines (654 and 663) coordinating the cluster were mutated to serines. Interestingly, upon elution from a Heparin HiTrap HP column by a gradient of sodium chloride, the mutant was eluted at a lower salt concentration compared to the intact hPolε_CD_ (SI Fig. S1). Heparin HiTrap is considered an affinity column for DNA-binding proteins because its sulfate groups mimic phosphates of DNA; therefore, the earlier mutant elution from this column indicates a compromised interaction with DNA. Of note, hPolε_CD_^M^ is contaminated with a proteolysis product of a molecular mass of ~ 120 kDa, due to reduced affinity to the Heparin column.

Purification of hPolε_CD_^M^ by a size-exclusion column revealed significant level of aggregates that were eluted in the void volume (SI Fig. S2). Of note, a slightly increased tendency to aggregate was mentioned for the yeast Polε_CD_ mutant deficient in the [4Fe-4S] cluster^10^. Thus, the size-exclusion column is an important step for hPolε_CD_^M^ purification, allowing removal of aggregated molecules. The concentrated hPolε_CD_^M^ sample was colorless, and almost iron-free (iron content of 2.8 ± 0.9%), as was previously shown for yeast Polε mutants^10,11^.

**Figure 3:**
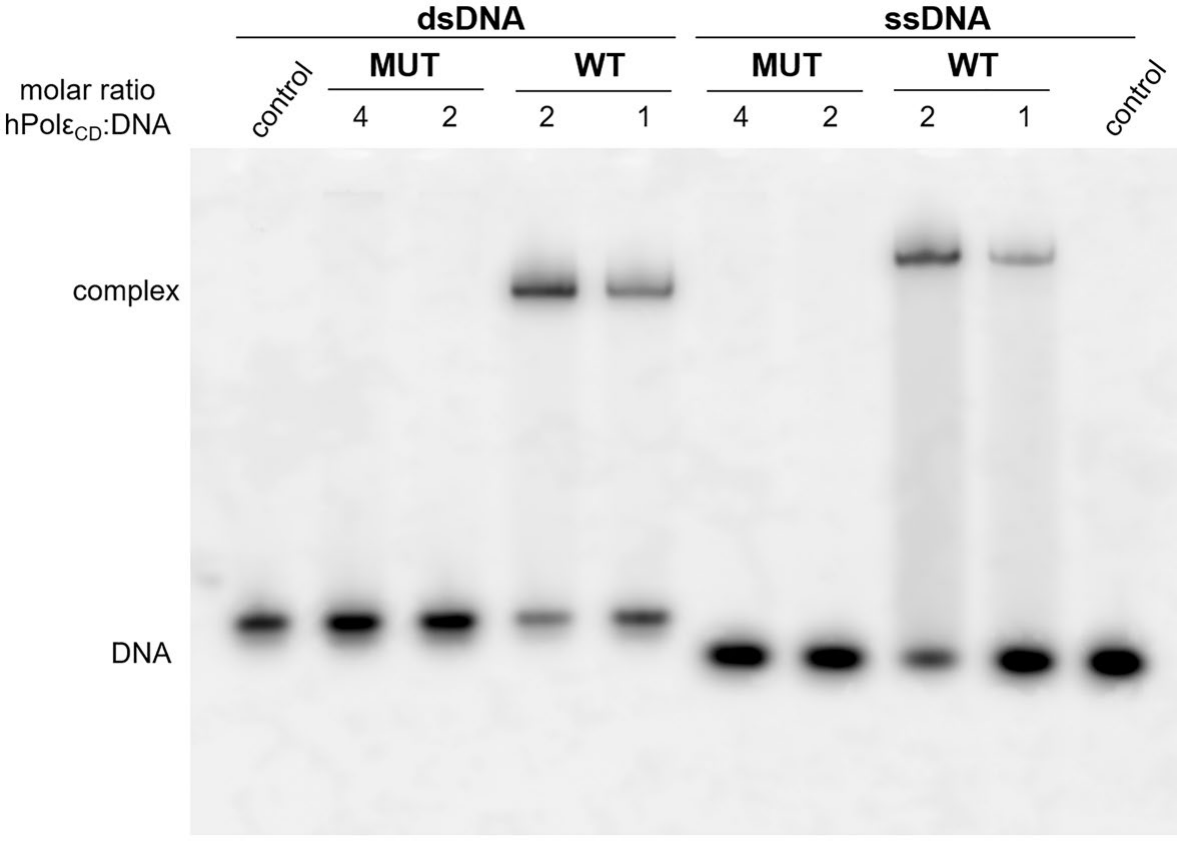
Effect of C654S/C663S mutation on DNA binding properties of hPolε_CD_. A 15-mer Cy3-labeled primer alone (ssDNA) or in the duplex with a 20-mer template (dsDNA) was incubated for 5 min with hPolε_CD_ (WT) and its mutant (MUT). The products were separated by electrophoresis in 5% acrylamide gel and visualized by Typhoon FLA 9500.

Analysis of the DNA-binding activity by EMSA revealed that the interaction with DNA is significantly compromised in the case of the mutant (Fig. 3). Even at four-fold molar excess over DNA, hPolε_CD_^M^ had no effect on the mobility of single-stranded (ss) and double-stranded (ds) DNA. In contrast, addition of hPolε_CD_ resulted in a significant mobility shift for both DNA substrates, with 72% of dsDNA and 51% of ssDNA in the complex with a protein added at two-fold molar excess (Fig. 3).

**Figure 4:**
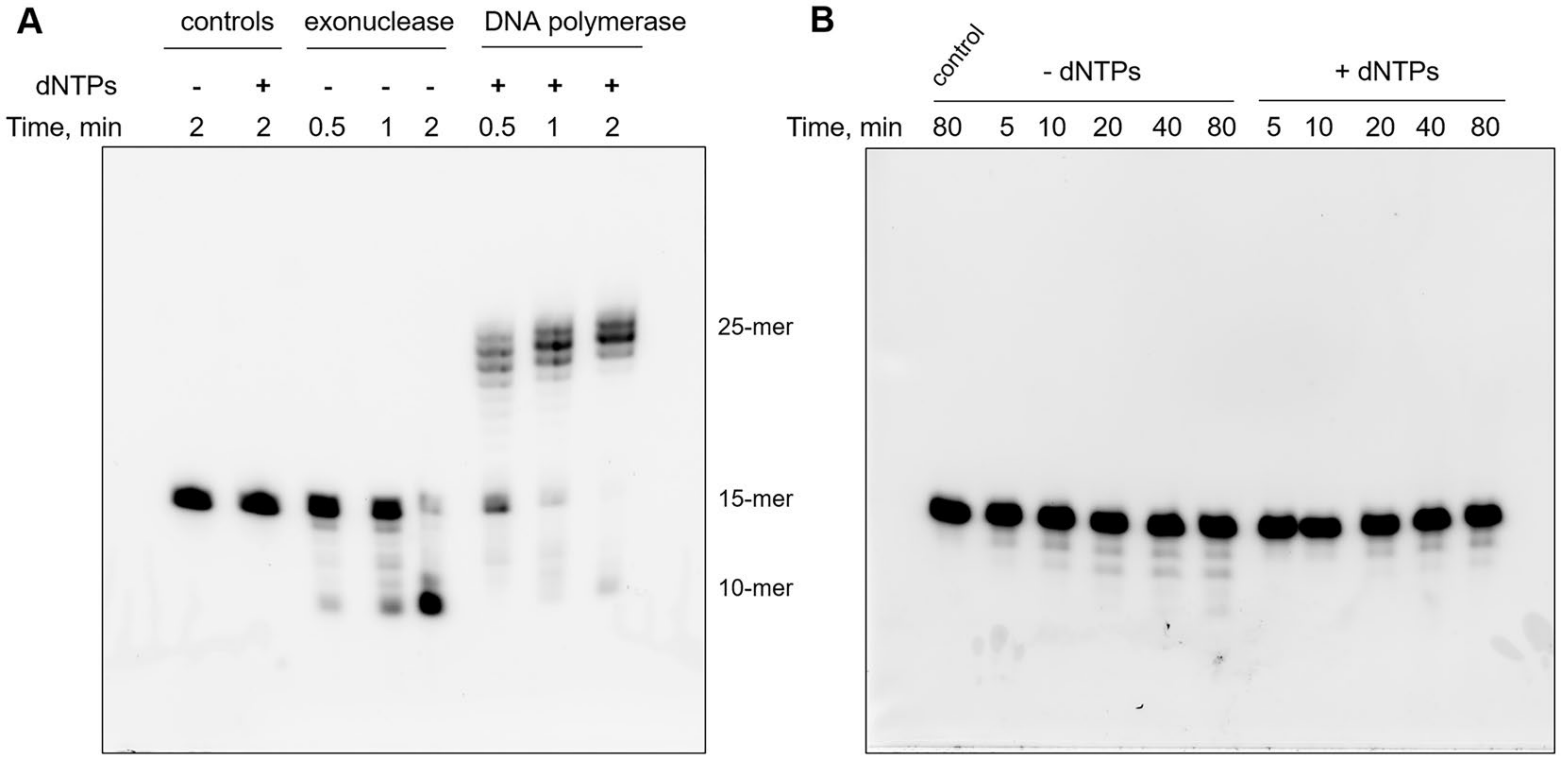
Analysis of DNA-polymerase and exonuclease activities of hPolε_CD_ (**A**) and hPolε_CD_^M^ (**B**). Primer extension assay was performed at 35 °C in the presence of dNTPs and a DNA substrate with a Cy3-labeled 15-mer primer annealed to a 25-mer template. Exonuclease activity was analyzed at similar conditions except the absence of dNTPs. The products were separated on 20% denaturing polyacrylamide gel and visualized by Typhoon FLA 9500.

Next, we analyzed the DNA polymerase and exonuclease activities of hPolε_CD_ and the mutant using a Cy3-labeled 15-mer primer annealed to a 25-mer template. Both activities require the presence of magnesium ions. In addition, a dNTP mix was added to the DNA polymerization reactions. Upon a 1–2 min incubation of the primed template with hPolε_CD_, almost all 15-mer primers were extended to longer products in the presence of dNTPs or degraded to smaller products in the absence of dNTPs (Fig. 4A). A disruption of the iron-sulfur cluster coordination by a C654S/C663S mutation dramatically affected exonuclease and especially DNA polymerase activity, which was not detected even after 80 min incubation at 35 °C (Fig. 4B).

**Figure 5:**
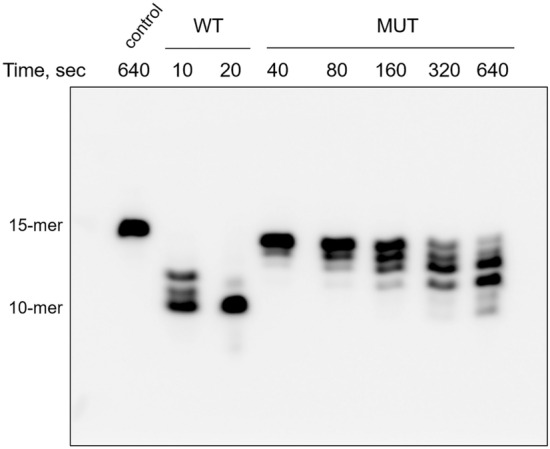
hPolε_CD_^M^ exhibits dramatically reduced exonuclease activity on single-stranded DNA. Exonuclease assay was performed at 35 °C using a 15-mer single-stranded DNA labeled with a Cy3 fluorophore at the 5′-end. The products were separated on 20% denaturing polyacrylamide gel and visualized by Typhoon FLA 9500.

Analysis of the exonuclease activity on the primer alone also showed that hPolε_CD_^M^ is significantly less active than the protein with an intact CysX motif (Fig. 5). It is known that DNA polymerases demonstrate stronger exonuclease activity on ssDNA versus dsDNA because only the primer 3′-end enters the exonuclease active site after partial duplex melting^11^. Thus, the absence of a [4Fe-4S] cluster severely affects DNA binding as well as DNA-polymerase and exonuclease activities of hPolε on the primed template.

### Binding and kinetic studies of PolεCD

The analysis of Polε_CD_ interaction with DNA was conducted using an Octet K2, which allows for extraction of the rate constants of complex formation (*k*_on_), dissociation (*k*_off_), and the dissociation constant (*K*_*D*_), which is inversely proportional to affinity. A 23-mer DNA template with biotin at the 5′-end (Table 2) was primed by an 11-mer DNA primer and loaded on a streptavidin-coated sensor. In the presence of 0.1 M NaCl, hPolε_CD_ samples obtained from *E. coli* and insect cells have shown similar *K*_*D*_ values close to 200 nM (Table 3). A previous DNA-binding study of hPolε_CD_ conducted in the absence of salt reported a *K*_*D*_ value of 79 nM ^16^.

**Table 3:** Interaction of hPolε_CD_ with DNA.

Expression system	[NaCl] mM	*k*_on_ mM^−1^ s^−1^	*k*_off_ × 10^–3^ s^−1^	*K*_*D*_^a^ nM
Insect	100	162 ± 2.5	34.2 ± 6.3	213 ± 43
*E. coli*	100	252 ± 26	41.9 ± 6.7	167 ± 24
*E. coli*	150	168 ± 29	542 ± 15	3258 ± 386

^a^ *K*_d_ values are obtained by dividing *k*_off_ by *k*_on_.

Data are presented as mean ± SD.

Notably, an increase of NaCl concentration from 0.1 to 0.15 M reduced affinity ~ 20-fold, which is mainly due to the 13-fold reduction in *k*_off_ value (Table 3). We observed a similar effect of salt concentration on affinity to DNA for Polα_CD_^17^ and for a different hPolε_CD_ construct, deficient in exonuclease activity^18^. Thus, hPolε_CD_ binds a DNA duplex with relatively low affinity at near-physiological salt concentration (Table 3, *K*_*D*_ = 3.3 µM). The obtained *k*_off_ value of 0.54 s^−1^ indicates that the half-life of the hPolε_CD_/DNA complex is ~ 1.3 s on average. It was not possible to conduct binding studies for hPolε_CD_ (insect) at 0.15 M NaCl with acceptable accuracy due to the low level of active molecules, which demands very high protein concentration in reaction.

**Figure 6:**
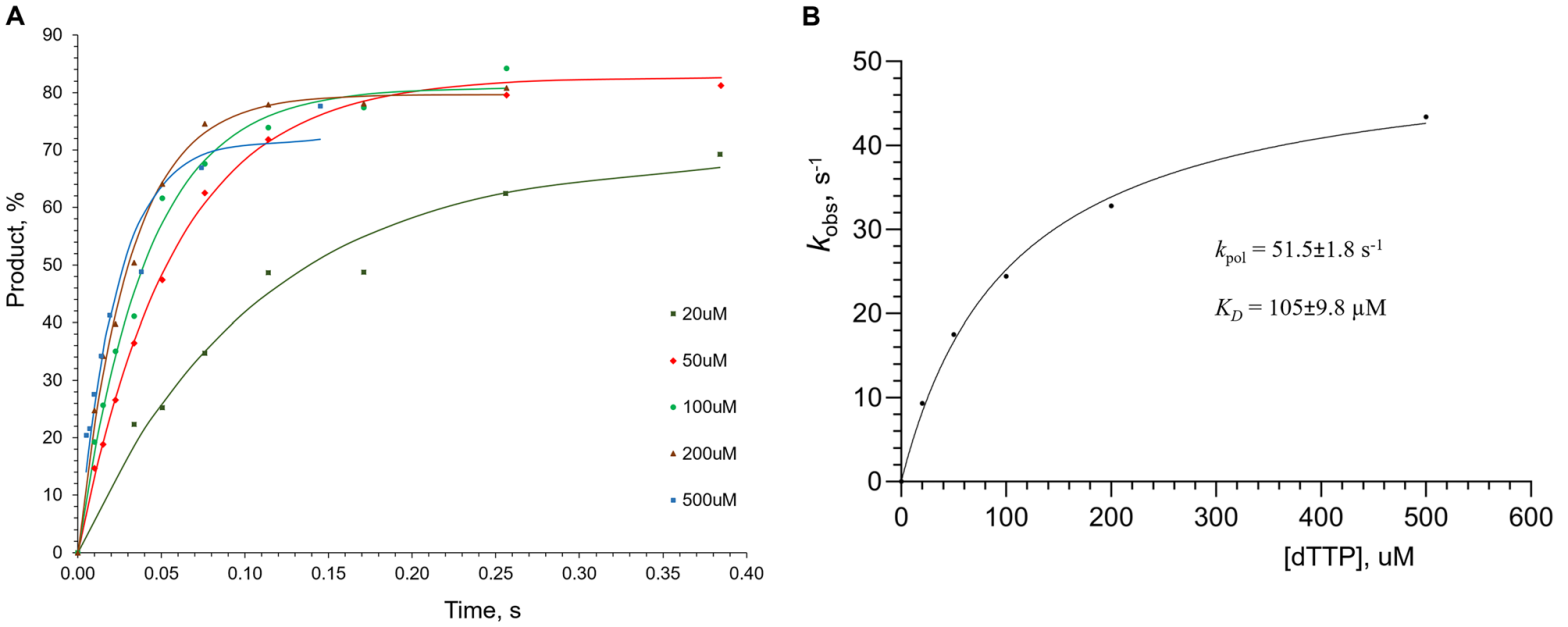
Single turnover kinetics of primer extension by hPolε_CD_. (**A**) Percent of extended primer was plotted against time and the data were fit to a single-exponential equation (Eq. 1). (**B**) Primer extension rates are plotted against dTTP concentration and the data were fit to a hyperbolic equation (Eq. 2) to obtain *K*_*D*_ and *k*_pol_ values. Reactions, containing 0.8 µM hPolε_CD_, 0.2 µM DNA, and dTTP at varied concentrations, were incubated at 35 °C at indicated time points.

The pre-steady-state kinetic approach was employed in order to assess hPolε_CD_ activity in DNA primer extension and its affinity to incoming dNTP at conditions used for binding studies. Kinetic studies were conducted in the presence of 0.1 M NaCl to allow most of the DNA to complex with hPolε_CD_ before the reaction. Single-nucleotide incorporation experiments were conducted under single-turnover conditions. This assay provides the maximal polymerization rate (*k*_pol_) and the apparent dissociation constant (*K*_*D*_) for the incoming nucleotide. hPolε_CD_ (0.8 µM) was incubated with a Cy3-labeled DNA (0.4 µM) and quickly mixed with varying dTTP concentrations under rapid chemical quench conditions. For each dTTP concentration, the fraction of extended primer was plotted against time (Fig. 6A) and the data were fit to a single-exponential equation (Eq. 1).

The obtained rate constant values were plotted against dTTP concentration (Fig. 6B) and fit to (Eq. 2), resulting in a *k*_pol_ of 51.5 ± 1.8 s^−1^ and an apparent *K*_*D*_ of 105 ± 9.8 µM. A *K*_*D*_ value of 31 µM was reported previously for the hPolε_CD_(exo-)/dTTP complex by another group^16^. The 3.4-fold difference with our data might be attributable to the absence of salt in reaction in that study. As shown above (Table 3), salt significantly affects the hPolε_CD_/DNA complex. The same effect is expected for the hPolε_CD_/dNTP complex because their interaction interface is mainly electrostatic. An apparent *K*_*D*_ of 105 µM for dTTP indicates that hPolε might not be saturated with dNTPs in vivo to achieve the maximal polymerization rate, given the fact that the average concentration of each dNTP in human mitotic cells is approximately 50 µM^19^ or even lower^20^. On the other hand, it is possible that the local concentration of dNTPs near the replication fork is significantly elevated. hPolε_CD_ (insect) was not suitable for this assay because of its low level of active molecules. In another study, we have shown that the exonuclease deficient variant of hPolε_CD_ extends the primer at a rate of 62.4 s^−1^ in the presence of 1 mM dTTP^18^. Interestingly, a *k*_pol_ value of 248 s^−1^ was obtained for hPolε_CD_ at 20 °C^16^, which might be due to the absence of salt in reaction^21^. Noteworthy, the catalytic domain of human Polα showed the maximal rate of DNA polymerization of 33.8 s^−1^ in the presence of 0.1 M NaCl^22^.

## Discussion

**Figure 7:**
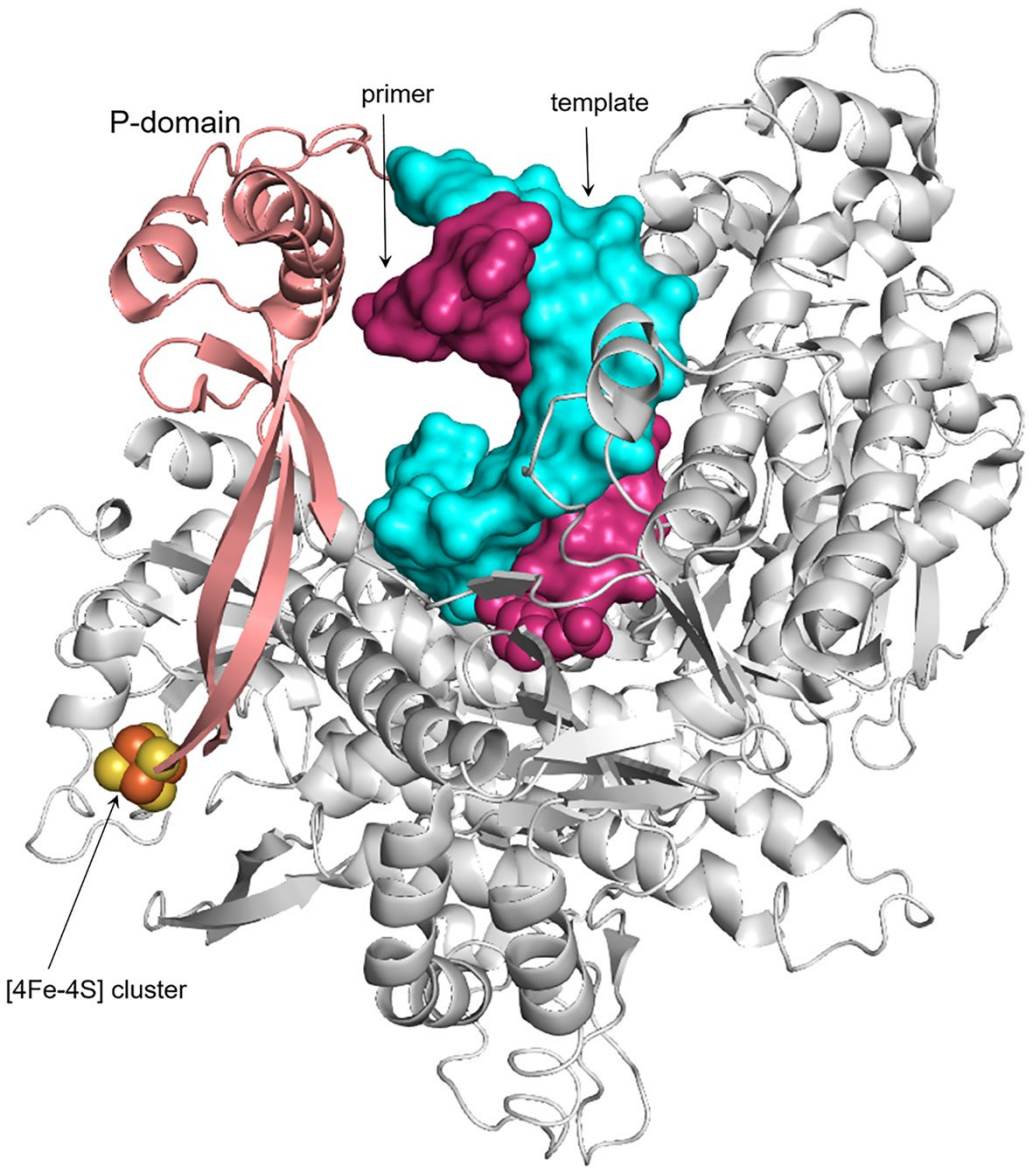
Position of the P-domain and the iron-sulfur cluster in yeast Polε. The P- and thumb domains and the rest of a protein are colored salmon, green, and gray, respectively. Template and primer are presented as surface and colored as cyan and pink, respectively. The 3′-terminal nucleotide of a primer is presented as spheres. The iron and sulfur in the [4Fe-4S] cluster are presented as spheres and colored brown and yellow, respectively. The crystal structure of a ternary complex of yeast Polε_CD_ with DNA and dATP (pdb code 6QIB^11^) was used for this presentation.

The recently discovered [4Fe-4S] cluster, located at the base of the P-domain (Fig. 7) and coordinated by four conserved cysteines, is unique to Polε^10,11^. Mutation of these cysteines in yeast Polε led to a loss of iron and DNA polymerase activity but not exonuclease activity, as was shown for holoenzyme as well as a separate catalytic domain^10,11^. The role of the [4Fe-4S] cluster in the DNA-binding properties of Polε has not been studied so far. Our data indicate that the iron-sulfur cluster is critical for interaction of human Polε with DNA. Probably, abrogated DNA binding is responsible for the loss of both activities in hPolε_CD_. Substrate binding by any enzyme is an initial step that precedes catalysis. It is difficult to imagine how the absence of the distantly located iron-sulfur cluster can affect both catalytic centers and especially the exonuclease one. Structural data indicate that the [4Fe-4S] cluster may stabilize the P-domain that helps Polε to enclose a DNA duplex (Fig. 7)^11^. This suggests that in the absence of the cluster, the P-domain changes its conformation and/or position and blocks entrance into the DNA-binding pocket for a template:primer.

It is quite possible that inactivation of DNA polymerase activity of yeast Polε by mutation of cluster-coordinating cysteines is also due to abrogated DNA binding and not catalysis. Intriguingly, the exonuclease activity of yeast Polε was not affected upon disruption of the [4Fe-4S] cluster^10,11^. Moreover, the DNA polymerase activity slightly recovered when dNTPs concentration was increased ten-fold, pointing to compromised dNTP binding^11^. We cannot exclude the possibility that elimination of the cluster has a slightly different effect on the structure and activity of yeast and human Polε. For example, in cluster-deficient mutants of yeast Polε, the template:primer-binding cleft might be blocked by the P-domain at the smaller level than in hPolε_CD_^M^.

The biological role of the [4Fe-4S] cluster in regulating DNA-binding properties and activity of Polε requires further investigation. As previously proposed^10^, due to its peripheral position and potential sensitivity to oxidation, the cluster might be a target for oxygen reactive species during oxidative stress. Recently it was demonstrated that the [4Fe-4S] cluster of yeast Polε is redox active and its reversible oxidation affects DNA polymerase activity^15^. We propose that the main role of the P-domain in hPolε is controlling access to the DNA-binding pocket, which might depend on intracellular signals. Even a subtle change in the P-domain position would be enough to prevent template:primer binding (Fig. 7). The concept of the interplay between the P-domain and the [4Fe-4S] cluster in the function of Polε is supported by the fact that they both exist only in the Polε catalytic domain and not in the catalytic domains of other DNA polymerases. On the other hand, we cannot exclude a possibility that the [4Fe-4S] cluster also controls or stabilizes the conformation of the other subdomains like fingers and even of the entire hPolε_CD_.

This work revealed that insect cells do not efficiently incorporate the [4Fe-4S] cluster into heterologous proteins during overexpression. This may be due to low capacity and/or high specificity of the enzymatic machinery, which is responsible for cluster incorporation. In contrast, the corresponding machinery of bacteria is more robust and non-specific. Previously we have shown that the [4Fe-4S] cluster was erroneously incorporated at a significant level into a zinc-binding domain located at the C-terminus of the catalytic subunit of hPolε^13,14^. Fortunately, the cluster-containing molecules were unable to make a stable complex with an accessory subunit of hPolε and were separated during purification. Thus, only the Polε catalytic domain contains the iron-sulfur cluster^10,11^. This study underscores the importance of measuring the iron level in Polε samples, especially when the enzyme is overexpressed in insect cells. Notably, all previous functional studies of hPolε_CD_ were conducted with a recombinant protein expressed in *E. coli*, where the iron content was not analyzed^16,23–25^.

## Materials and methods

### Cloning, expression, and purification

hPolε_CD_ was cloned into pASHSUL-1 plasmid^26^ to produce the corresponding proteins tagged with N-terminal His-Sumo. The mutant with cysteines 654 and 663 changed to serines was obtained by side-directed mutagenesis. hPolε_CD_(insect) with a cleavable N-terminal His-Tev tag was cloned into pFastBac-1 plasmid (Invitrogen). hPolε_CD_ and hPolε_CD_(exo-) were expressed in *E. coli* strain Rosetta-2 (DE3) at 18 °C for 16 h following induction with 0.2 µg/ml anhydrotetracycline. Afterward, cells were harvested by centrifugation at 4000 g for 15 min, washed with PBS, aliquoted, and kept at −80 °C. A high-titer virus stock for hPolε_CD_(insect) was obtained by using the Bac-to-Bac Baculovirus Expression System from Invitrogen. 1.8 × 10^9^ Sf21 cells in 1 L shaking culture were infected with the recombinant virus at a multiplicity of infection of 2 and cultivated at 27 °C for 56 h. Cells were harvested by centrifugation at 200 g for 5 min and frozen.

All samples were purified according to the same protocol, including chromatography on a Ni-IDA column (Bio-Rad), His-tag digestion during overnight dialysis, and chromatography on a Heparin HP HiTrap column (Cytiva) and on a size-exclusion column Superose 12 10/300 GL (Cytiva) in buffer containing 25 mM Tris-HEPES (pH 7.8), 0.15 M NaCl, 1% glycerol, and 2 mM tris(2-carboxyethyl)phosphine (TCEP). Finally, samples were concentrated to 30–60 µM and flash-frozen in aliquots. The purity of obtained samples was analyzed by 8% SDS-PAGE (Fig. 1). Protein concentrations were estimated by measuring the absorbance at 280 nm and using extinction coefficients of 157 mM^−1^ cm^−1^; the extinction coefficients were calculated with ProtParam^27^. The iron content in purified protein samples was determined with use of chromogen ferrozine as described in^22^.

#### Electrophoretic mobility shift assay

Reactions containing 0.5 µM DNA (Table 2) and varying amount of protein were incubated in 10 μl for 5 min at room temperature in buffer containing 20 mM Tris-Hepes (pH 7.8), 100 mM NaCl, 2% glycerol, 2 mM TCEP, and 0.2 mg/mL BSA; 5 μl was then loaded on 5% native PAGE. Samples labeled with Cy3-dye were visualized using a Typhoon 9410 imager (Cytiva) and quantified using ImageJ software (version 1.45 s, National Institutes of Health).

#### Binding studies

Analysis of binding kinetics was done at 23 °C on an Octet K2 (Sartorius AG). This device uses Bio-Layer Interferometry technology to monitor molecular interactions in real time. A template with a biotin-TEG at the 5′-end was annealed to the primer (Table 2) and immobilized on a streptavidin-coated biosensor (SAX, Sartorius AG). The primer was added at two-fold molar excess over the template and contained 3'-dideoxy cytidine. SAX sensors were loaded with oligonucleotide-biotin at 50 nM concentration for 7 min at 500 rpm. Then sensors were blocked by incubating for 2 min in 10 µg/ml biocytin. In the first row of a 96-well microplate (Greiner Bio-One), the first six wells contained the buffer, consisting of 30 mM Tris-Hepes, pH 7.8, 100 mM or 150 mM NaCl, 2 mM TCEP, and 0.002% Tween 20. The next six wells contained the two-fold dilutions of hPolε_CD_ in the same buffer. All wells in the adjacent row contained only the buffer for reference. Data Analysis HT software (ver. 11.1, Sartorius AG) was used for calculation of binding constants (*k*_on_, *k*_off_, and *K*_*D*_) by using the global fitting. The average value and standard deviation were calculated from three independent experiments.

#### Pre-steady-state kinetic studies

Kinetic studies were performed on a QFM-4000 rapid chemical quench apparatus (BioLogic, France) at 35 °C. Reactions contained 0.4 µM hPolε_CD_ (active molecules), 0.2 µM DNA, varying concentrations of dTTP, 25 mM Tris-HEPES, pH 7.8, 0.1 M NaCl, 8 mM MgCl_2_, 2 mM TCEP, and 0.2 mg/mL BSA. hPolε_CD_ was incubated with a Cy3-labeled 15-mer primer annealed to a 25-mer DNA template (Table 2), to allow formation of the binary complex, and rapidly mixed with dTTP and MgCl_2_ followed by quenching with 0.3 M EDTA. Products were collected in a tube containing 15ul 100% formamide and separated by 20% Urea-PAGE. The Cy3-labeled products were visualized by a Typhoon FLA 9500 (Cytiva) and quantified by ImageJ, version 1.5.3 (NIH). The extended primer fraction was calculated by dividing the amount of extended primer by the amount of primer added in reaction. For each dTTP concentration, the percent of extended primer was plotted against time and the data were fit to a single exponential equation:

1 $$\left[ {{\text{product}}} \right] \, = A\times (1 \, {-}e^{{{-}kobst}} )$$

where *A* is the amplitude, *k*_*obs*_ is the observed rate for dNTP incorporation, and *t* is the time. The *k*_*obs*_ was plotted against dTTP concentration and the data were fit to the hyperbolic equation:

2 $${k}_{\mathrm{obs}}=\frac{{k}_{pol} \times \left[\mathrm{dNTP}\right]}{{K}_{D}+ \left[\mathrm{dNTP}\right]}$$

using GraphPad Prizm software to obtain *k*_*pol*_, the maximum rate of nucleotide incorporation, and *K*_*D*_, the apparent dissociation constant for the incoming nucleotide.

#### DNA polymerase and exonuclease assay

Exonuclease reactions were conducted in 10 µl at 35 °C and contained 0.05 µM hPolε_CD_ or its mutant (total protein concentration), 0.2 µM DNA (Cy3-labeled 15-mer primer annealed to a 25-mer DNA template), 25 mM Tris-HEPES, pH 7.8, 0.1 M NaCl, 5 mM MgCl_2_, 2 mM TCEP, and 0.2 mg/mL BSA. DNA polymerase reactions were performed in a similar way except using 10 nM protein and adding 50 µM dNTPs. The reactions were stopped by addition of 20 µl stop solution (96% formamide, 50 mM EDTA, 0.1% bromophenol blue) and heated to 95 °C for 1 min. The products were separated on a 20% denaturing polyacrylamide gel and visualized by a Typhoon FLA 9500 (Cytiva).

## Supplementary Information

Supplementary Information.

## Data Availability

The data that support the findings of this study are included in the Supplementary Information file or available from the corresponding author on request.

## Abbreviations

hPolε: Human DNA polymerase ε
hPolε_CD_: Catalytic domain of human DNA polymerase ε
P-domain: Processivity domain
EMSA: Electrophoretic mobility gel shift assay
TCEP: Tris(2-carboxyethyl)phosphine

## References

[1] Lujan SA, Williams JS, Kunkel TA (2016). DNA polymerases divide the labor of genome replication. Trends Cell Biol..

[2] Hogg M, Johansson E (2012). DNA polymerase epsilon. Subcell. Biochem..

[3] Henninger EE, Pursell ZF (2014). DNA polymerase epsilon and its roles in genome stability. IUBMB Life.

[4] Tahirov TH, Makarova KS, Rogozin IB, Pavlov YI, Koonin EV (2009). Evolution of DNA polymerases: An inactivated polymerase-exonuclease module in Pol epsilon and a chimeric origin of eukaryotic polymerases from two classes of archaeal ancestors. Biol. Direct..

[5] Dua R, Levy DL, Campbell JL (1999). Analysis of the essential functions of the C-terminal protein/protein interaction domain of Saccharomyces cerevisiae pol epsilon and its unexpected ability to support growth in the absence of the DNA polymerase domain. J. Biol. Chem..

[6] Zhou JC, Janska A, Goswami P, Renault L, Abid Ali F, Kotecha A, Diffley JFX, Costa A (2017). CMG-Pol epsilon dynamics suggests a mechanism for the establishment of leading-strand synthesis in the eukaryotic replisome. Proc. Natl. Acad. Sci. U S A.

[7] Yuan Z, Georgescu R, Schauer GD, O'Donnell ME, Li H (2020). Structure of the polymerase epsilon holoenzyme and atomic model of the leading strand replisome. Nat. Commun..

[8] Pachlopnik Schmid J, Lemoine R, Nehme N, Cormier-Daire V, Revy P, Debeurme F, Debre M, Nitschke P, Bole-Feysot C, Legeai-Mallet L, Lim A, de Villartay JP, Picard C, Durandy A, Fischer A, de Saint Basile G (2012). Polymerase epsilon1 mutation in a human syndrome with facial dysmorphism, immunodeficiency, livedo, and short stature ("FILS syndrome"). J. Exp. Med..

[9] Bellelli R, Borel V, Logan C, Svendsen J, Cox DE, Nye E, Metcalfe K, O'Connell SM, Stamp G, Flynn HR, Snijders AP, Lassailly F, Jackson A, Boulton SJ (2018). Polepsilon instability drives replication stress, abnormal development, and tumorigenesis. Mol. Cell..

[10] Jain R, Vanamee ES, Dzikovski BG, Buku A, Johnson RE, Prakash L, Prakash S, Aggarwal AK (2014). An iron-sulfur cluster in the polymerase domain of yeast DNA polymerase epsilon. J. Mol. Biol..

[11] Ter Beek J, Parkash V, Bylund GO, Osterman P, Sauer-Eriksson AE, Johansson E (2019). Structural evidence for an essential Fe-S cluster in the catalytic core domain of DNA polymerase. Nucl. Acids Res..

[12] Netz DJ, Stith CM, Stumpfig M, Kopf G, Vogel D, Genau HM, Stodola JL, Lill R, Burgers PM, Pierik AJ (2011). Eukaryotic DNA polymerases require an iron-sulfur cluster for the formation of active complexes. Nat. Chem. Biol..

[13] Baranovskiy AG, Lada AG, Siebler HM, Zhang Y, Pavlov YI, Tahirov TH (2012). DNA polymerase delta and zeta switch by sharing accessory subunits of DNA polymerase delta. J. Biol. Chem..

[14] Baranovskiy AG, Gu J, Babayeva ND, Kurinov I, Pavlov YI, Tahirov TH (2017). Crystal structure of the human Pol B-subunit in complex with the C-terminal domain of the catalytic subunit. J. Biol. Chem..

[15] Pinto MN, Ter Beek J, Ekanger LA, Johansson E, Barton JK (2021). The [4Fe4S] cluster of yeast DNA polymerase epsilon is redox active and can undergo DNA-mediated signaling. J. Am. Chem. Soc..

[16] Zahurancik WJ, Klein SJ, Suo Z (2013). Kinetic mechanism of DNA polymerization catalyzed by human DNA polymerase epsilon. Biochemistry.

[17] Baranovskiy AG, Babayeva ND, Lisova AE, Morstadt LM, Tahirov TH (2022). Structural and functional insight into mismatch extension by human DNA polymerase alpha. Proc. Natl. Acad. Sci. U S A.

[18] Lisova AE, Baranovskiy AG, Morstadt LM, Babayeva ND, Tahirov TH (2022). The incoming dNTP makes human DNA polymerase ε discriminative against RNA- containing primers. Sci. Rep..

[19] Ferraro P, Franzolin E, Pontarin G, Reichard P, Bianchi V (2010). Quantitation of cellular deoxynucleoside triphosphates. Nucl. Acids Res..

[20] Coggins SA, Mahboubi B, Schinazi RF, Kim B (2020). Mechanistic cross-talk between DNA/RNA polymerase enzyme kinetics and nucleotide substrate availability in cells: Implications for polymerase inhibitor discovery. J. Biol. Chem..

[21] Chui G, Linn S (1995). Further characterization of HeLa DNA polymerase epsilon. J. Biol. Chem..

[22] Baranovskiy AG, Duong VN, Babayeva ND, Zhang Y, Pavlov YI, Anderson KS, Tahirov TH (2018). Activity and fidelity of human DNA polymerase alpha depend on primer structure. J. Biol. Chem..

[23] Goksenin AY, Zahurancik W, LeCompte KG, Taggart DJ, Suo Z, Pursell ZF (2012). Human DNA polymerase epsilon is able to efficiently extend from multiple consecutive ribonucleotides. J. Biol. Chem..

[24] Eddy S, Maddukuri L, Ketkar A, Zafar MK, Henninger EE, Pursell ZF, Eoff RL (2015). Evidence for the kinetic partitioning of polymerase activity on G-quadruplex DNA. Biochemistry.

[25] Zahurancik WJ, Suo Z (2020). Kinetic investigation of the polymerase and exonuclease activities of human DNA polymerase epsilon holoenzyme. J. Biol. Chem..

[26] Weeks SD, Drinker M, Loll PJ (2007). Ligation independent cloning vectors for expression of SUMO fusions. Protein Expr. Purif..

[27] Gasteiger E, Hoogland C, Gattiker A, Duvaud S, Wilkins MR, Appel RD, Bairoch A, Walker JM (2005). Protein identification and analysis tools on the ExPASy server. The proteomics protocols handbook.

