# Supplementary material for: The iron-sulfur cluster is essential for DNA binding by human DNA polymerase ε
Source: Sci Rep. 2022 Oct 19;12:17436. doi: 10.1038/s41598-022-21550-4 (PMC9581978; doi:10.1038/s41598-022-21550-4)
Supplement: Supplementary file 1 — Supplementary Information. [file 41598_2022_21550_MOESM1_ESM.pdf]

SUPPLEMENTAL INFORMATION

for the article

The iron-sulfur cluster is essential for DNA binding by human DNA polymerase ε

Alisa E. Lisova[1a], Andrey G. Baranovskiy[1a], Lucia M. Morstadt[1], Nigar D. Babayeva[1], Elena I. Stepchenkova[1b], Tahir H. Tahirov[1]*

Tahir H. Tahirov
Email: ttahirov@unmc.edu

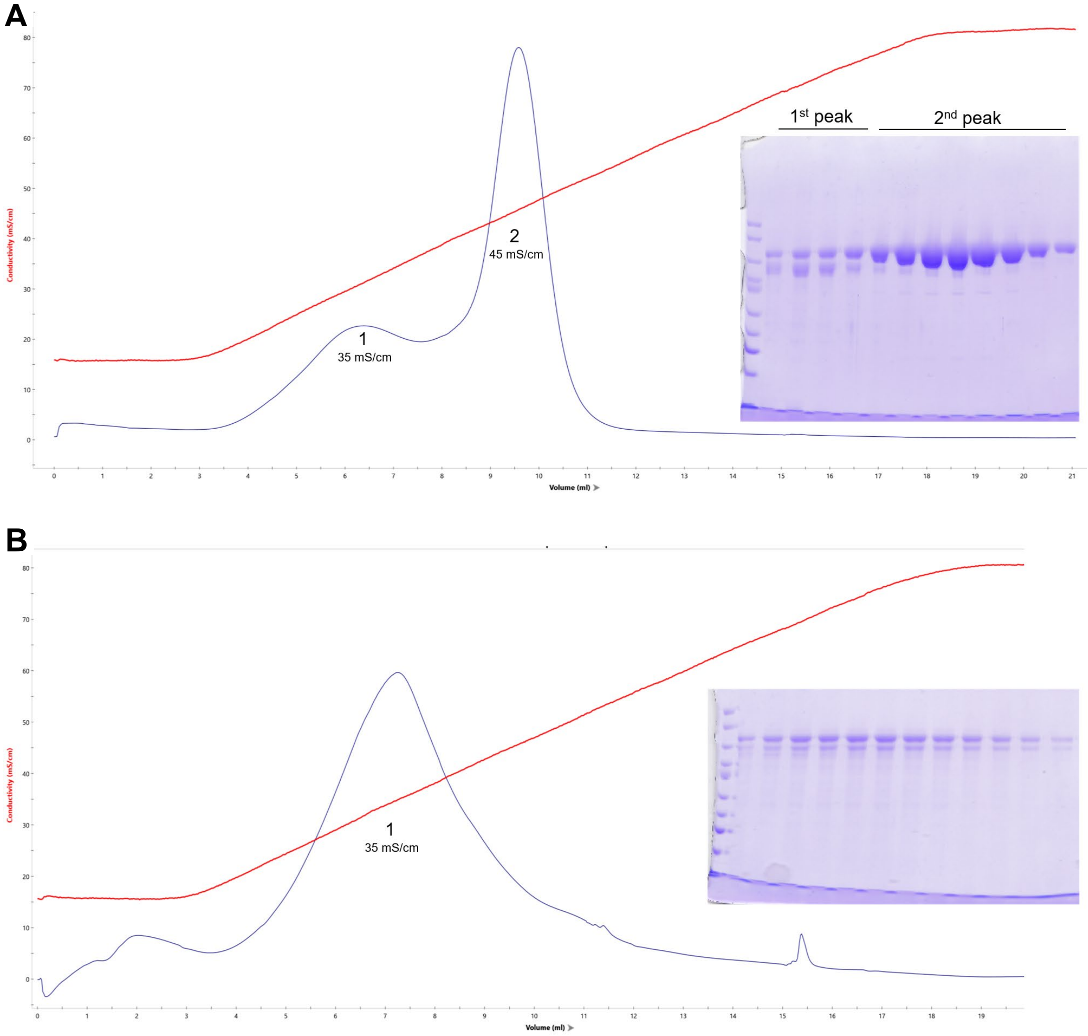

Figure S1. Heparin elution profiles of hPolε_{CD} (A) and hPolε_{CD}^{M} (B). Heparin HiTrap HP column (Cytiva, 1 ml) was eluted by 15 ml gradient of 0.15 - 0.8 M NaCl. Peak fractions were analyzed by 8% SDS-PAGE and stained by Coomassie Brilliant Blue R-250. The mutant elutes as a wide peak and at lower salt concentration than wild-type hPolε_{CD}, resulting in contamination with a proteolyzed form.

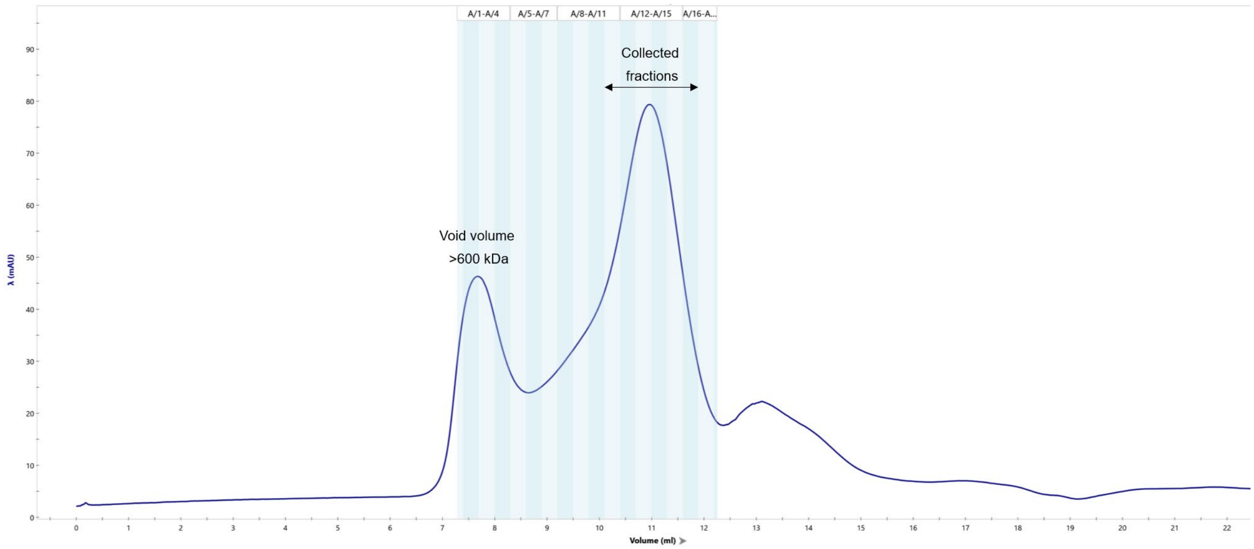

Figure S2. Elution profile of hPolε$_{CD}$^{M} on Superose 12 size exclusion column. Superose 12 10/300 GL (Cytiva) has a bed volume of 24 ml and an exclusion limit of 300 kDa for globular proteins. The chromatography was conducted at 4ºC in the buffer containing 25 mM Tris-HEPES (pH 7.8), 0.15 M NaCl, 1% glycerol, and 2 mM TCEP.
